# The potential impact of the Comprehensive and Progressive Agreement for Trans-Pacific Partnership on Thailand’s hepatitis C treatment program

**DOI:** 10.1186/s12992-024-01053-9

**Published:** 2024-06-13

**Authors:** Brigitte Tenni, Joel Lexchin, Chutima Akaleephan, Chalermsak Kittitrakul, Deborah Gleeson

**Affiliations:** 1 School of Psychology and Public Health, La Trobe University, Melbourne, Australia; 2 Nossal Institute for Global Health, University of Melbourne, Melbourne, Australia; 3 School of Health Policy and Management, York University, Toronto, Canada; 4 International Health Policy Programme, Ministry of Public Health, Bangkok, Thailand; 5 AIDS Access Foundation Bangkok, Bangkok, Thailand

**Keywords:** Intellectual property, Patents, Generic medicines, Compulsory licencing, TRIPS flexibilities, Trade agreements, TRIPS-plus, Hepatitis C, Direct-acting antivirals

## Abstract

**Background:**

Thailand has expressed interest in joining the Comprehensive and Progressive Agreement for Trans-Pacific Partnership (CPTPP), a twelve-country plurilateral trade agreement whose original incarnation included the United States of America (USA). When the USA withdrew from this agreement, key intellectual property clauses relevant to pharmaceuticals were suspended. These could be reinstated should the CPTPP Parties decide to do so.

**Methods:**

This study uses two scenarios to cost the impact the CPTPP would have had on Thailand’s 2020 hepatitis C treatment regime if Thailand joined the CPTPP and suspended clauses were reinstated.

**Results:**

Joining the CPTPP could have increased the cost more than tenfold if suspended CPTPP clauses were reinstated and Thailand was not willing or able to issue compulsory licenses. Based on the 2020 budget, the price for this possible scenario could have reduced hepatitis C treatment coverage by 90%.

**Conclusions:**

Acceding to trade agreements such as the CPTPP that require increasing intellectual property protection, could compromise Thailand’s hepatitis C program and other national treatment programs reliant on affordable generic medicines. The CPTPP could also prevent Thailand from relying on its own pharmaceutical capabilities to manufacture medicines needed to sustain its treatment programs.

**Supplementary Information:**

The online version contains supplementary material available at 10.1186/s12992-024-01053-9.

## Introduction and background

Thailand has repeatedly expressed interest in joining the Comprehensive and Progressive Agreement for Trans-Pacific Partnership (CPTPP) [1], a plurilateral trade agreement between Australia, Brunei Darussalam, Canada, Chile, Japan, Malaysia, Mexico, Peru, New Zealand, Singapore, Vietnam and the United Kingdom (UK). This research aims to measure the potential impact that accession to this agreement could have had on access to medicines in Thailand in 2020, specifically the impact of its suspended intellectual property (IP) provisions on access to direct acting antivirals (DAA) used to treat hepatitis C.

As a Member of the World Trade Organization (WTO), Thailand must comply with its Agreement on Trade-Related Aspects of Intellectual Property Rights (TRIPS) and provide minimum standards of IP protection, including making patents available for least 20 years from the time the application is filed, for pharmaceutical products or processes that meet standard criteria for patentability, novelty, inventive step and industrial applicability [2]. While these provisions impose obligations to make patents available for pharmaceuticals, TRIPS also contains “flexibilities” or provisions that can be used by Member States to mitigate the impact of patents, such as excessively high medicine prices, in the interests of public health.

Together with Ecuador, Thailand has made the most frequent use of compulsory licencing of any country [3]. Compulsory licenses are an important example of TRIPS flexibilities [4]. A compulsory licence is an authorisation granted by a government that allows the government or a third party to produce a patented product or to use a patented process without the consent of the patent holder [4]. The patent holder must be paid adequate remuneration that takes into account the economic value of the authorisation [5]. In 2006 to 2008, Thailand issued a total of seven government public non-commercial use compulsory licences for HIV, cardiovascular and cancer medicines [6–8].

While many other countries have signed bilateral and/or regional trade agreements that commit them to IP protections that go beyond those required by TRIPS, or that limit their use of TRIPS flexibilities, Thailand has not yet done so. However, this situation could change if Thailand joins the CPTPP.

### Thailand and the Comprehensive and Progressive Agreement for Trans-Pacific Partnership

The Trans-Pacific Partnership Agreement (TPP), the precursor to the CPTPP, was signed in February 2016, and had not entered into force when President Donald Trump withdrew the United States (US) in January 2017 [9]. The TPP included an IP chapter with multiple provisions expected to have a negative impact on access to medicines [10]. After the US withdrawal, several TRIPS-plus IP provisions were suspended but could be reintroduced if the member countries agree to end suspension of one or more of these provisions [11]. The suspended provisions are explained in Table 1.

**Table 1:** Suspended TRIPS-plus IP articles of the CPTPP

Suspended article	Explanation
Article 18.37 (Patentable Subject Matter) (i) paragraph 2: all of this paragraph; (ii) paragraph 4: the last sentence;	Patents to be made available for at least one of: new uses of a known product, new methods of using a known product or new processes of using a known product. Patents must be available for inventions derived from plants.
Article 18.46 (Patent Term Adjustment for Unreasonable Granting Authority Delays): all of this Article including footnotes 36 through 39;	Adjust, upon request, a patent’s term of protection to compensate the patent owner if there are unreasonable delays in a patent office’s issuance of a patent.
Article 18.48 (Patent Term Adjustment for Unreasonable Curtailment): all of this Article including footnotes 45 through 48;	Adjust a pharmaceutical patent’s term of protection to compensate the patent owner for unreasonable curtailment of the effective term of a patent as a result of the marketing approval process for a pharmaceutical product.
Article 18.50 (Protection of Undisclosed Test or Other Data): all of this Article including footnotes 50 through 57;	A period of at least 5 years during which a regulator cannot provide marketing approval for a generic version that relies on the original clinical trial data submitted to the regulatory agency to prove safety and efficacy of a new medicine.
Article 18.51 (Biologics): all of this Article including footnotes 58 through 60;	At least eight years of effective market protection for biologics, provided via at least 8 years of data exclusivity or at least 5 years of data exclusivity and other measures to ‘deliver a comparable outcome in the market’.

Adapted from Comprehensive and Progressive Agreement for Trans-Pacific Partnership text. See https://www.dfat.gov.au/sites/default/files/tpp-11-treaty-text.pdf

The remaining 11 countries negotiated and signed a new trade agreement called the CPTPP in March 2018, which incorporates most of the provisions of the TPP [12]. In July 2023, the UK signed an Accession Protocol to join the agreement after two years of accession negotiations with the existing parties [12] – the same process that would be followed by Thailand if it formally requested accession and the other parties agreed to admit it.

Membership in the CPTPP is seen by many in the Thai government as a demonstration of international relevance and a conduit to joining future trade agreements as well as an opportunity to boost exports, attract foreign direct investment, and create jobs [13]. Critics of the CPTPP have decried the potential impact of the CPTPP on access to medicines due its TRIPS-plus provisions that exceed the minimum standards for IP protection and enforcement required by the TRIPS Agreement, although most of these provisions have been suspended [14, 15].

If some or all of the suspended clauses are reinstated in the future all member countries would need to ensure their domestic legislation is compliant. Most of the higher-income countries in the CPTPP already have provisions in their patent law that reflect these suspended clauses. The impact of these currently suspended provisions would therefore be felt most keenly in lower-income countries which are already the least able to provide affordable access to medicines for their populations [16].

It is important to note, however, that the CPTPP IP chapter does not place additional restrictions on compulsory licensing. Article 18.6 of the TPP, retained in the CPTPP, reaffirms each party’s right to take measures to protect public health and to use TRIPS flexibilities [17]. The final text of the data exclusivity provision of the TPP included an exception (Article 18.50 paragraph 3, which also applies to Article 18.51 for biologics) for measures to protect public health in accordance with the Declaration on TRIPS and Public Health [18]. This provision means CPTPP signatories should be able to incorporate exceptions from data exclusivity for compulsory licensing in their domestic laws without breaching the CPTPP even if the data exclusivity provisions are reinstated.

While the CPTPP IP chapter does not create additional obstacles to compulsory licensing, it is possible that its investment chapter may. IP rights are recognised as covered investments in Article 9.1 of the Investment Chapter, and investors may use the CPTPP’s investor-state dispute settlement (ISDS) mechanism to initiate arbitration in an international tribunal when they believe their investor rights under the agreement have been breached [19]. Article 9.8 paragraph 5 provides an exception to the expropriation provisions for compulsory licenses that are compliant with the CPTPP IP chapter and the TRIPS Agreement, but it is possible that this (as yet untested) exception may not be sufficiently robust, or that an ISDS case over compulsory licensing could be initiated using the investment chapter’s provisions for ‘fair and equitable treatment’ (Article 9.6) [19]. Scholars have raised concerns about the robustness of several exceptions in the Investment Chapter, including the exception in Annex 9-B paragraph 3(b) which excludes ‘non-discriminatory regulatory actions…that are designed and applied to protect legitimate public welfare objectives, such as public health, safety and the environment…’ from claims regarding indirect expropriation, ‘except in rare circumstances’ [14, 20].

Provisions in other CPTPP chapters have implications for various aspects of pharmaceutical policy [21], however these are beyond the scope of the current paper, which focuses on the impact of the CPTPP’s IP provisions.

Thailand’s national hepatitis C program was chosen to examine the potential impact of the CPTPP on access to medicines, should Thailand choose to become a member, for several reasons. Hepatitis C remains a significant health burden in Thailand. To address this issue requires considerable scale up of testing and treatment. Thailand has been providing free hepatitis C treatment in the government health care system since 2018 and has the industrial capacity to manufacture generic DAAs used to treat hepatitis C making treatment more affordable and sustainable. It was therefore important to examine whether increased IP protections introduced as a result of membership in the CPTPP, could hamper the local manufacture or importation of generic DAAs.

### Hepatitis C treatment and Thailand’s 2020 hepatitis C treatment regime

In the past decade, the introduction of DAAs has revolutionized hepatitis C treatment, demonstrating high efficacy and good tolerability. Previous treatment for hepatitis C had consisted of injectable pegylated interferon alpha and oral ribavirin. This treatment was less efficacious than the DAAs and took up to a year [22] compared with 8–24 weeks with the use of DAAs (see Table 2). Side effects in some people were severe and commonly resulted in default from treatment [23].

**Table 2:** WHO hepatitis C treatment guidelines for adults 2018

Hepatitis C positive patients	Treatment combination	Treatment duration
Patients > 18 years without cirrhosis	Glecaprevir/Pibrentasvir	8 weeks*
	Sofosbuvir/Daclatasvir	12 weeks
	Sofosbuvir/Velpatasvir	12 weeks
Patients > 18 years with compensated cirrhosis	Glecaprevir/Pibrentasvir	12 weeks*
	Sofosbuvir/Daclatasvir	24 weeks
	Sofosbuvir/Daclatasvir	12 weeks**
	Sofosbuvir/Velpatasvir	12 weeks

* Persons with hepatitis C genotype 3 infection who have received interferon and/or ribavirin in the past should be treated for 16 weeks

** May be considered in countries where genotype distribution is known and genotype 3 prevalence is < 5%

Adapted from the WHO hepatitis C treatment guidelines 2018

The hepatitis C virus (HCV) has six genotypes named 1 to 6. The World Health Organization (WHO) recommends therapy with pan-genotypic DAAs (i.e., those that can be used for all genotypes) for persons over the age of 12 years [24]. DAAs can cure 90% or more of people with HCV infection and treatment duration is short (usually 8 to 24 weeks), depending on the absence or presence of cirrhosis [24]. WHO treatment guidelines for adults are detailed in Table 2.

Sofosbuvir (SOF), a DAA produced by Gilead Sciences (hereafter, Gilead) and marketed as Sovaldi, was first launched in 2014 at a price that made it unaffordable to most people even in high-income countries. A 12-week combination treatment with a sofosbuvir-based regimen ranged from US$84,000 to $94,000 or approximately US$1000 per pill [25]. In its first year on the market, Sovaldi generated $US10.3 billion in sales for Gilead and came close to being the highest revenue producing medicine in the world [26]. In contrast, a study from 2014 estimated the manufacturing cost of a 12-week course of sofosbuvir to be US$68–US$136 [27].

Approximately 760,000–790,000 people in Thailand are HCV seropositive and about half are living with chronic infection [28, 29]. Although genotype 1 is the most prevalent in the western world [30], genotype 3 is most common in Thailand at 46.1%, followed by genotypes 1, 6 and 2 at 32.5%, 20.9%, and 0.5%, respectively [31]. Due to cost and resource constraints, DAAs were not available for several years to all people living with HCV in Thailand and treatment was limited to a small number of people with chronic HCV due to stringent eligibility criteria [32], with some older peg-interferon and ribavirin-based therapies being used [33]. Treatment regimens were not pan-genotypic and therefore required genotype testing which adds to the treatment costs. These regimens are detailed in Table 3.

**Table 3:** Thai government reimbursement scheme for hepatitis C treatment regimens in 2020

Hepatitis C genotype	Treatment regime	Length of treatment
3	SOF + Peg-IFN + RBV combination therapy	12 weeks
1, 2, 4, and 6 without liver cirrhosis	SOF + LDV combination therapy	12 weeks
1, 2, 4, and 6 with liver cirrhosis	SOF + LDV + RBV combination therapy	12 weeks

Adapted from Sirinawasatien A, Techasirioangkun T. Sofosbuvir-based regimens in the treatment of patients with chronic hepatitis C virus infection: Real-world efficacy in Thailand. PLoS One. 2020 Feb 27;15 [2]:e0229517

SOF = Sofosbuvir, LDV = Ledipasvir, RBV = Ribavirin, Peg-IFN = Pegylated interferon

Thailand’s 2020 hepatitis C treatment regime included SOF and SOF/ledipasvir (LDV) sourced from the Indian generic company, Mylan. Ribavirin and peg-interferon were sourced from Merck Sharp & Dohme and Roche respectively [33]. Many patent applications have been filed for DAAs in Thailand. All patent applications are pending and many patents for DAAs, including SOF, have been opposed by patient groups and NGOs on the grounds that they lack novelty and an inventive step [34]. The details of the DAA patent applications in Thailand are listed in Supplementary File 1.

## Aim

This study aims to estimate the impact the CPTPP would have had on Thailand’s 2020 hepatitis C program if it had joined the CPTPP and the suspended clauses had been reinstated. Impact is measured as the cost of the treatment program and the number of people able to access treatment.

## Methods

### Scenario development

Two scenarios were designed to explore the potential impact of joining the CPTPP on Thailand’s hepatitis C treatment program:

- Scenario 1 (baseline): This scenario involves Thailand’s patent law in 2020, the 2020 hepatitis C treatment regime and no issuance of compulsory licenses for hepatitis C medicines. In this scenario, Thailand does not join the CPTPP and the suspended clauses are not reinstated.
- Scenario 2: In this scenario, Thailand joins the CPTPP, suspended clauses are reinstated, and the 2020 hepatitis C treatment regime is used. No compulsory licenses are issued for hepatitis C medicines.

The study focuses on the hepatitis C program in 2020 for several reasons. Thailand expressed interest in joining the CPTPP at that time (and since) and was providing free hepatitis C treatment in the government health care system using DAAs and some older peg-interferon and ribavirin-based therapies due to resource constraints. The treatment program in that year provided an appropriate baseline scenario against which to measure the potential impact of the CPTPP suspended provisions. In addition, the National Health Security Office (NHSO) was able to provide price and treatment data for the complete 2020 year. Data for the hepatitis C treatment program in later years were not available at the time of writing.

To finalise Scenario 2 and its assumptions, it was necessary to determine if the CPTPP and its suspended clauses would require legislative change in Thailand, should they be reinstated. Relevant Thai IP laws and policies were examined for compliance with patent linkage^1^ and CPTPP suspended IP clauses. See Table 1 for an explanation of the clauses.

To establish whether patents would be granted for the DAAs used in the 2020 treatment regime under Scenario 2, the Medspal^2^ website was searched for patents, patent applications and patent applicants for SOF and SOF/LDV. The cost of the DAAs^3^ in the Thai hepatitis C program was then calculated for the two scenarios given the prices outlined in the data collection. See Table 4 for the scenarios and their related assumptions.

**Table 4:** Scenarios for costing DAA in hepatitis C treatment program

	Scenario 1 - Baseline – 2020 treatment regime	Scenario 2- Thailand joins CPTPP (suspended clauses are reinstated)
**Regime**	• SOF/LDV for genotype 1,2,4,6 • SOF for genotype 3 • Genotype test	• SOF/LDV for genotype 1,2,4,6 • SOF for genotype 3 • Genotype test
**Assumptions**	- Generic DAA regimes used in Thailand in 2020; - Uses Mylan generic price for Thailand in 2020; - Treatment for genotypes 1,2,4,6 use the DAA regime of SOF/LDV; - Treatment of people with HCV-3 is with SOF and Pegylated interferon and Ribavirin combination therapy. Only the SOF (DAA) component of this is costed; - Thailand does not join the CPTPP and the suspended clauses are not reinstated; - No compulsory licences are issued.	- Assumes patents are granted for all filed secondary patent applications as CPTPP allows for secondary patenting. (Patents to be made available for at least one of: new uses of a known product, new methods of using a known product or new processes of using a known product); - Patent terms may be extended due to CPTPP patent term extensions; - Data exclusivity and patent linkage may further delay marketing approval and/or market entry for generics; - Treatment for genotypes 1,2,4,6 use the DAA regime of SOF/LDV; - Treatment of people with HCV-3 is with SOF and Pegylated interferon and Ribavirin combination therapy. Only the SOF (DAA) component of this regime is costed; - No compulsory licences are issued.

SOF = Sofosbuvir, LDV = Ledipasvir

If Thailand chooses to make patents available for new uses only, then this scenario would not be valid as the only indication for the DAAs included in this scenario is for the treatment of hepatitis C at this time. The scenario is valid if Thailand chooses to make patents available for new methods of using a known product and or new processes of using a known product as that would result in secondary patents being granted for the DAAs in this scenario

The scenarios above do not include hepatitis C treatment for people with liver cirrhosis as the regime differed from the standard regime and the cost data for that regime was not available. It is expected that excluding this small group of people would not affect the overall results significantly.

### Data collection

The number of people treated with DAAs for hepatitis C in Thailand in 2020 was sourced from the Thai NHSO and was a constant for Scenarios 1 and 2. The price data for Scenario 1 was sourced from NHSO. Thailand was accessing SOF for $US293 and SOF/LDV for $US349 per course per person from Mylan. Price data for Scenario 2 and genotype testing prices were obtained from the Health Intervention and Technology Assessment Program (HITAP) study by Rattanavipapong et al.: “Revisiting policy on chronic HCV treatment under the Thai Universal Health Coverage: An economic evaluation and budget impact analysis”^5^ [35]. According to Rattanavipapong et al., Gilead offered Thailand a price of $US4,028.00 per course per person of SOF and $US5,323.00 per course per person of SOF/LDV in 2020.

### Data analysis

For each scenario people were divided into those treated with SOF/LDV for genotype 1,2,4,6 and those treated with SOF for genotype 3. For each scenario, the total cost of DAAs in the hepatitis C treatment was calculated by multiplying the cost of the relevant DAA regime (including the genotype test) per person by the number of people being treated for the year 2020. The total cost of each scenario was then calculated and compared. The number of people able to be treated based on the cost of the DAAs in Scenario 2 and the budget of Scenario 1 was determined by calculating the average per person cost of treatment in Scenario 2 and then dividing the total cost of Scenario 1 by this average to estimate the number of people able to be treated. The reduction in treatment coverage was calculated by assuming the treatment coverage in Scenario 1 (baseline) to be 100%. The number of people able to be treated in Scenario 2 was then calculated as a percentage (n%) of the number of people treated in Scenario 1 or conversely as the percentage drop in treatment coverage (100%-n%).

## Results

Scenario 1 is the baseline policy option available to Thailand and does not require legislative change. Scenario 2 would require legislative change as outlined in Table 5. Joining the CPTPP would allow for patents to be made available for at least one of: new uses of a known product, new methods of using a known product or new processes of using a known product (see the assumptions in Table 4). Scenario 2 assumes patents are available for new methods and or new processes of using a known product and therefore the pending new methods patent applications for SOF and SOF/LDV by Gilead could be granted. Thailand would then be compelled to pay the prices set by Gilead, the patent holder of these DAAs.

**Table 5:** CPTPP suspended clauses and corresponding legal changes required in Thailand

CPTPP suspended clauses and existing patent linkage clause	Corresponding laws and regulations that would require reform
Article 18.37: Patentable Subject Matter Suspend Paragraph 2 and Paragraph 4, second sentence	Thai Patent Act Sect. 9. The following inventions are not protected under this Act: [4] **methods of diagnosis, treatment or cure of human and animal diseases;**
Article 18.46: Patent Term Adjustment for Unreasonable Granting Authority Delays Article 18:48: Patent Term Adjustment for Unreasonable Curtailment	Thai Patent Act Part III Rights Conferred by the Patent 35 [13]. An invention patent shall **have a term of twenty years from the date of filing of the application in the country**.
Article 18.50: Protection of Undisclosed Test or Other Data	Thai Trade Secret Act B.E.2558
Article 18.51: Biologics	**Thai Trade Secret Act B.E 2558**
Article 18.53: Patent Linkage	Thai Drug Act (No. 6) B.E. 2562 and its Regulation on marketing approval process

Adapted from Comprehensive and Progressive Agreement for Trans-Pacific Partnership text and Thai legal text. See https://www.dfat.gov.au/sites/default/files/tpp-11-treaty-text.pdf

The full cost of each scenario is detailed in Table 6.

**Table 6:** Cost of Direct Acting Antivirals in hepatitis C treatment in each scenario in US$

	Cost of DAA regime/test per person (US$)	Number of people treated	Total cost of DAA regime/genotyping for number of people treated
**Scenario 1**			
SOF/LDV for genotype 1,2,4,6	349	1993	695,557
SOF for genotype 3	293	1372	401,996
Genotyping	161.4	3365	543,111
Pharmaceutical company producing medication	Mylan		
Total cost of DAA in hepatitis C treatment	**1,640,664**		
**Scenario 2**			
SOF/LDV for genotype 1,2,4,6	5323	1993	10,608,739
SOF for genotype 3	4028	1372	5,526,416
Genotyping	161.4	3365	543,111
Pharmaceutical company producing medication	Gilead		
Total cost of DAA in hepatitis C treatment	**16,678,266**		
Average cost per person for Scenario 2	16,678,266/3365=$4956.39		
Number of people able to be treated given the cost of the regimes in scenario 2 and the budget of scenario 1.	1,640,644/4,956.39 = 331 people		
% treatment coverage due to the cost of scenario 2	331/3365 = 10% coverage of Scenario 1		
Drop in treatment coverage given the cost of the regimes in Scenario 2 and the budget of Scenario 1	90%		

SOF = Sofosbuvir, LDV = Ledipasvir

In Scenario 1 (the Baseline Scenario), Thailand was accessing SOF for $US293 and SOF/LDV for $US349 per course which was far cheaper than the $US4,028.00 per course of SOF and $US5,323.00 per course of SOF/LDV that Gilead offered Thailand in 2020. The total cost of Scenario 2 (Thailand joins CPTPP and suspended clauses are reinstated) is $US16,678,266 and is the more expensive scenario and more than ten times the total cost of the Scenario 1 (baseline DAA treatment program) at $US1,640,664. Within the 2020 budget, the cost of Scenario 2 would have reduced the number of people able to access treatment from 3,365 to 331, a drop in treatment coverage of 90%.

## Discussion

The cost of Scenario 2 demonstrates the potential implications for the cost of DAAs if Thailand had joined the CPTPP in or prior to 2020 and the suspended clauses had been reinstated at this time. The implications of this cost increase are multiple. Thailand would have had to choose whether to reduce the number of people being treated for hepatitis C or spend more than ten times the 2020 budget to treat the same number of people. Delaying hepatitis C treatment can lead to cirrhosis, end-stage liver disease, hepatocellular carcinoma and liver-related death [36] and adds to the suffering of people living with hepatitis C. The risk of developing cirrhosis for those with untreated chronic hepatitis C infection is between 15 and 30% within 20 years after infection [28]. Treatment for cirrhosis and hepatocellular carcinoma, another consequence of hepatitis C infection, is costly and complex and can include surgery and even liver transplantation [29]. Delaying DAA treatment could, therefore, add significant costs to Thailand’s universal health coverage program. Studies across various geographic regions including Thailand, have found early treatment for hepatitis C with low cost generic DAAs to be a cost-effective treatment and prevention strategy [30–33]. Additionally, a previous study that measured the impact of a TRIPS-plus trade agreement on the price of ARV and DAAs, found that data exclusivity and patent term extension clauses would lead to higher costs to the government [32].

### CPTPP and secondary patenting

The CPTPP has a number of suspended clauses that could have negative implications for the cost of DAAs, however, the most immediate impact would be due to suspended Article 18.37 (as outlined in Table 1) which allows for secondary patenting. When referring to medicines, a secondary patent is a patent on an aspect of a medicine other than the original active drug ingredient, such as a chemical variant, a new formulation or a new method of administration [34]. It can have the effect of extending the effective patent life of a medicine. One study found secondary patenting to extend the patent life of a medicine by six to seven years [37]. Another study found that secondary patents could extend patent life by 12 years beyond the patent expiration for the medicine’s base compounds and 39 years after the first patents on the medicine [34].

Thailand’s Patent Act allows for pre-grant opposition which offers third parties an opportunity to oppose the grant of a patent within 90 days of the patent application [38]. To successfully prevent a patent from being granted, they must demonstrate that the application does not meet patentability requirements. TPP Article 18.37, if reinstated in the CPTPP, would facilitate the granting of secondary DAA patents currently pending and opposed by patient groups. See Supplementary File 2 for a list of primary and secondary patent applications for SOF, LDV and VEL in Thailand. The list of secondary patent applications includes, but is not limited to, ‘sofosbuvir crystalline forms and preparation processes’ and ‘sofosbuvir processes and intermediates’. Grounds for opposition would be narrowed if patentability standards were lowered to allow patents for new uses of a known product, new methods of using a known product or new processes of using a known product. The availability of new use patents allows for additional patents to be granted for a medicine if it is found to be useful in the treatment of a different condition. New method patents allow for the patenting of reformulations of an existing patented medicine, for example, new dosing and new methods of administration. If Thailand chose to implement the ‘new uses’ instead of the ‘new methods’ option it may not impact on the patent status of DAAs, however, it is very likely to have a negative impact on access to other generic medicines.

### CPTPP and patent term extensions

Suspended Articles 18.46 and 18.48 allow for patent term extensions due to unreasonable delays in a patent office’s issuance of a patent or delays in the marketing approval process. If CPTPP membership required Thailand to incorporate these articles into their IP laws and policies, it could lengthen patent terms and delay the availability of affordable generic medicines. Although the impact of these clauses was not directly measured by this study, it is likely that patent term extensions could add to DAA treatment costs at a later stage. Studies of the impact of patent term extensions have found that they can maintain high treatment costs over time [39–41].

### CPTPP and threshold for inventiveness

The CPTPP text also includes a footnote to Article 18.37 paragraph 1 which establishes a lower threshold for inventiveness than is required by TRIPS:

*30 For the purposes of this Section, a Party may deem the terms ‘inventive step’ and ‘capable of industrial application’ to be synonymous with the terms ‘non-obvious’ and ‘useful’, respectively. In determinations regarding inventive step, or non-obviousness, each Party shall consider whether the claimed invention would have been obvious to a person skilled, or having ordinary skill in the art, having regard to prior art. (CPTPP Chap. 18, Footnote 30)*

This provision, particularly the reference to a person “having ordinary skill in the art” (which is TRIPS-plus) can result in the granting of poor-quality patents [42]. Although the Thai Patent Act (No.3) B.E. 2542 includes a similarly low inventiveness threshold, Thailand currently has the policy space to lift the threshold. Membership of the CPTPP would remove this element of discretion, creating a binding obligation to retain the low inventiveness threshold. This retention could result in patents being granted that would otherwise be rejected, such as the patent applications that are currently pending for DAAs (see Supplementary file 2). The outcome of this footnote to Article 18.37 is reflected in Scenario 2.

### CPTPP and patent linkage

Not all the TPP’s TRIPS-plus provisions were suspended in the CPTPP. For example, the CPTPP retained TPP Article 18.53 which provides for patent linkage. The CPTPP allows for two different approaches to patent linkage. The first, described in Art. 18.53 para 1, requires a system to alert the rights holder when a third party is seeking to market a product covered by a patent, and the provision of a judicial or administrative process for resolving disputes. The second alternative (Art 18.53 para 2) requires direct coordination between the marketing approval authority and the patent office, and an automatic stay on marketing approval where a patent may be infringed. The two options allow Thailand some flexibility in implementing patent linkage in a way that mitigates its impact on the market entry of generics. However, a health impact assessment found that implementing patent linkage could negatively affect the domestic pharmaceutical industry in Thailand by slowing the market entry of locally produced generic medicines. This change would increase Thailand’s reliance on more expensive imported medicines [43]. If Thailand joins the CPTPP, this impact could occur regardless of whether the suspended TRIPS-plus provisions are reinstated.

### CPTPP and voluntary licensing

Some commentators argue that potential patent barriers, like those created by membership of TRIPS-plus agreements such as the CPTPP, can be addressed by introducing remedial policies like voluntary licencing [44]. A voluntary licence is an authorisation given by a patent holder to a generic company, allowing it to produce a generic version of a patented pharmaceutical product [45]. Voluntary licences have been lauded as a practical way to overcome the barriers that patents pose such as high prices and can substantially improve access to HCV treatment in countries included in such agreements by improving the affordability and supply of generic products [46]. They have, however, been criticised for their lack of transparency and restrictive conditions that can undermine access to medicines [45]. When a patent is granted, a country’s ability to access an affordable generic can be contingent on the terms and conditions of the voluntary licence in play [47]. It is therefore fitting to explore the DAA voluntary licence landscape as it applies to Thailand to determine whether voluntary licencing can overcome the patent barriers that membership of the CPTPP may impose.

The United Nations-backed Medicines Patent Pool (MPP) works to facilitate voluntary licenses for priority medicines and pool IP to encourage generic manufacture and the development of new medicine formulations [48]. It has signed agreements with three patent holders for three hepatitis C DAAs; daclatasvir ((DAC)- Bristol Myers Squibb (BMS)) glecaprevir/pibrentasvir ((G/P)- AbbVie) and ravidasvir ((RAV)- Pharco)) [49]. These licences are detailed in Supplementary File 1. All three DAA MPP licences exclude Thailand and many other middle-income countries which account for the majority of the global burden of hepatitis C infection. The BMS and the AbbVie MPP Agreements allow countries not included in the licence to be supplied by MPP licensees provided no patent is being infringed. However, as there are no Thai DAA patents, Thailand is unencumbered in seeking the best generic price on the global market which may or may not include those from the MPP licensees. Scenario 1 demonstrates that voluntary licensees do not necessarily provide the least expensive generic option.

Thailand was also excluded from Gilead’s 2014 voluntary licence that licenced the manufacture of SOF directly to 11 generic manufacturers in India [50]. It was only after Malaysia made use of TRIPS flexibilities and issued a compulsory licence for SOF that Gilead extended its bilateral licence in August 2017 to include Thailand and several other high burden middle-income countries [45]. The compulsory licence allowed Malaysia to import a generic at a cheaper price than the generic companies included in the voluntary licence offered [51].

Although Gilead’s bilateral voluntary licence appears broad and far-reaching it only allows for eleven Indian, one Pakistani and two Egyptian generic companies to produce and sell SOF [52]. Thailand has an established pharmaceutical manufacturing industry with the capacity to manufacture a range of pharmaceuticals including DAAs. The Government Pharmaceutical Organization (GPO) is a wholly government-owned enterprise that has long supplied the majority of antiretrovirals for the national HIV treatment program [53]. Limiting the bilateral licence to include only Indian generic companies would have prohibited Thailand from using its own production capacity which produces medicines that are less expensive and less reliant on global supply chains. The aforementioned bilateral licence created a pseudo-monopoly for Indian companies included in the licence and empowered them to charge non-competitive prices especially if only one of the companies decided to register their product in a country, as was the case with Thailand. It is unclear and somewhat perplexing as to why Thailand paid $US349 for a course of SOF/LDV treatment and $US293 for a course of SOF despite the bilateral agreement in place and the absence of patents on DAAs. This price was significantly more than the lowest priced generic SOF available globally in 2020 at $US60 and $US150 for a course of pan-genotypic SOF/VEL [47]. The rationale for this procurement choice warrants further explanation but is beyond the scope of this study.

Currently, as there are no patents granted for DAAs in Thailand, it is not locked into paying high prices for patented DAAs nor is it confined to the terms of Gilead’s bilateral licence. It was also using a superseded regime (pegylated interferon and ribavirin) that is no longer recommended by WHO, has a longer treatment duration, is less efficacious and is associated with significant side effects. Membership of the CPTPP could limit Thailand’s options to access affordable generics compared to the prices it was paying due to the existing imperfect DAA voluntary licences.

### Thailand’s current DAA regime

Treatment with a generic pan-genotypic regime is recommended by WHO and was an option for Thailand in 2020. Exercising this option would have negated the expense and complication of genotype testing. It is the least expensive, most effective treatment option with the fewest side effects available to Thailand. Thailand has provided this option since January 2021 using sofosbuvir and velpatasvir (SOF/VEL) generics from Mylan, although access was reported to be complicated by eligibility criteria, requirements for pre-treatment diagnostic tests, and the need for specialist administration of HCV treatment [54]. The GPO obtained marketing approval for its generic SOF/VEL in October 2022 which has allowed for local production and supply [54]. Thailand has now adopted a pan-genotypic treatment regime ensuring great self-sufficiency and cost-effectiveness, however the ability to continue to produce this product could be impacted if a patent is granted for SOF/VEL. Patents for SOF/VEL have been filed by Gilead and opposed by patient groups.

### Future options for the Thai Hepatitis C treatment program

If Thailand joined the CPTPP, it could be locked into paying high prices for patented DAAs and or restrict Thailand to buying from Indian generic companies included in bilateral pharmaceutical voluntary licences due to compliance with the CPTPP IP chapter and suspended clauses. This arrangement would compromise its purchasing power and restrict Thailand from producing its own DAAs, which it has the industrial capacity to do. Joining the CPTPP would not restrict Thailand’s ability to issue a compulsory licence to access cheaper generics and so this remains a viable option, however it is possible that Thailand could face ISDS disputes over compulsory licenses brought using the ISDS mechanism provided for in the Investment Chapter of the CPTPP. Although no ISDS cases have been brought or threatened on this basis, foreign investors have used ISDS to challenge a country’s IPR legal system. For example, ISDS clauses were used by Philip Morris to challenge Australia’s tobacco plain packaging laws and by Eli Lilly to challenge a Canadian patent ruling. According to Prabhash Ranjan, “the possibility of pharmaceutical companies challenging the issuance of CL before ISDS tribunals is real, not conjectural” [55].

Compulsory licensing is often an onerous and time-consuming process and comes with its own challenges. Thailand faced enormous backlash from the USA and the pharmaceutical company Abbott Laboratories when it issued seven compulsory licences in the past. Following the licences, the USA placed Thailand on the United States Trade Representative (USTR) Priority Watch List under Special 301^6^ of the Omnibus Trade and Competitiveness Act of 1988. It also threatened to rescind the trade privileges granted to Thailand under the Generalised System of Preferences [56]. Abbott refused to register any new medicines in Thailand and withdrew any medicines it had awaiting registration [57]. These past actions may make Thailand hesitant to pursue compulsory licenses for DAAs should it become necessary to do so.

### Limitations

The Scenarios did not calculate the full cost of treatment, only the cost of the DAA component. The 2020 Thai treatment regime for genotype 3, the most common HCV genotype in Thailand, required treatment with DAAs and pegylated interferon and ribavirin on the basis of cost. However, this treatment is no longer recommended by the WHO and has been superseded by pan-genotypic DAA. Therefore, as the treatment costs of Scenarios 1 and 2 calculated the DAA component only and did not include the pegylated interferon and ribavirin, these costs are likely to be underestimated in comparison to the costs of a pan-genotypic treatment regime.

In contrast to retrospective studies, prospective studies of the impact of trade agreements tend to find larger negative effects of stronger IP provisions on prices and costs of medicines [58]. As this is a prospective analysis, it is possible that assumptions made in this study overestimate the likely impact of the CPTPP.

In addition, this cross-sectional study measured only the most immediate impacts of the CPTPP if Thailand joined and the suspended clauses were reinstated. Some of the suspended CPTPP TRIPS-plus provisions and the patent linkage provision could be expected to have more cumulative effects over time and their impact on the price of medicines would not be realised for years to come [59]. Further research is needed to better understand the impact of patent linkage, patent term extensions and data exclusivity.

It is possible that Thailand will join the CPTPP, and the suspended clauses are not reinstated in their original form. This scenario was not costed in the study due to the difficulty of estimating the potential impacts of patent linkage provisions.

The results of this study are limited to the impact on DAAs only and findings are not necessarily generalisable to other medicines. Additionally, this study only analyses the impact of the IP provisions and not the full set of trade rules in the CPTPP.

## Conclusion

This study adds to the scant literature that has quantified the potential impact of a TRIPS-plus trade agreement on access to DAAs, a class of medicines that has been fraught with patent barriers and high prices. If Thailand had joined the CPTPP in or prior to 2020 and its suspended TRIPS-plus clauses had been reinstated, the cost of the 2020 DAA treatment program could have increased more than ten times over. A cheaper option for Thailand than its 2020 treatment regime, and one it has since adopted, is to use a locally produced generic pan-genotypic regime. This is currently a viable option because no DAA has been granted a patent to date. However, the ability of Thailand to produce this pan-genotypic generic DAA would have been compromised had it signed the CPTPP. Thailand is at a critical juncture with regard to CPTPP membership. It needs to consider the broader implications of joining the CPTPP including the potential impact on the price of medicines if the suspended IP clauses are reinstated. Failing to heed the risks that TRIPS-plus agreements pose could threaten not only the sustainability and expansion of its hepatitis C program but also other national treatment programs reliant on affordable generic medicines. Signing trade agreements that contain TRIPS-plus measures could threaten its ability to draw on its own pharmaceutical capabilities to manufacture the medicines needed to sustain its treatment programs. The potential impact of the loss of manufacturing autonomy needs to be taken into account when governments make decisions about trade agreements. This study provides a warning to countries about the potential consequences of ignoring IP provisions in trade agreements.

### Electronic supplementary material

Below is the link to the electronic supplementary material.

Supplementary Material 1

## Data Availability

All data generated or analysed during this study are included in this published article and its supplementary information files.

## Footnotes

1 This is a conditional relationship between the granting of marketing approval for a generic medicine and the patent status of the originator medicine.

2 MedsPaL is a medicines patent and licences database administered by the Medicines Patent Pool. It provides information on the patent and licensing status of selected HIV, hepatitis C, tuberculosis and other patented essential medicines in low- and middle-income countries.

3 The costs of ribavirin and pegylated interferon were not included in the calculations. This decision was made because they are old, superseded medicines whose patents have expired in the vast majority of the world. The price of ribavirin and pegylated interferon would have been a constant for Scenarios 1 and 2 and therefore would not have changed the results in terms of how the cost of these Scenarios compare to one another.

5 Some prices in this study were in Thai baht - these were converted into USD at 31.28 baht per USD – the average exchange rate for US$/Thai baht for 2020.

6 The USTR Special 301 Report is an annual review of the global state of IP protection and enforcement relevant to US interests. As a consequence of being placed on the Priority Watch Lists, the US government can eliminate tariff preferences unilaterally granted, such as the Generalized System of Preferences (GSP). They can also initiate dispute settlement proceedings at the WTO or through other existing trade agreements.

## Abbreviations

ARV: Antiretroviral (medication)
BMS: Bristol-Myers Squibb
CPTPP: Comprehensive and Progressive Agreement for Trans-Pacific Partnership
DAA: Direct acting antiviral (medication)
DAC: Daclatasvir
G/P: Glecaprevir/pibrentasvir
GPO: Government Pharmaceutical Organization
HCV: Hepatitis C virus
IP: Intellectual property
ISDS: Investor-state dispute settlement
LDV: Ledipasvir
NHSO: National Health Security Office (NHSO
Peg-IFN: Pegylated interferon
RAV: Ravidasvir
RBV: Ribavirin
SOF: Sofosbuvir
TPP: Trans-Pacific Partnership Agreement
TRIPS: Agreement on Trade-Related Aspects of Intellectual Property Rights
UK: United Kingdom
USA: United States of America
VEL: Velpatasvir
WHO: World Health Organization
WTO: World Trade Organization
