# Supplementary material for: The potential impact of the Comprehensive and Progressive Agreement for Trans-Pacific Partnership on Thailand’s hepatitis C treatment program
Source: Global Health. 2024 Jun 13;20:46. doi: 10.1186/s12992-024-01053-9 (PMC11170909; doi:10.1186/s12992-024-01053-9)
Supplement: Supplementary file 1 — Supplementary Material 1 [file 12992_2024_1053_MOESM1_ESM.docx]

**Supplementary File 1: Abbvie and Gilead DAA Patent Applications in Thailand**

| **Product Name(s) and Patent applicant** | **Patent Description** | **Patent Status** |  |
| --- | --- | --- | --- |
| Abbvie Glecaprevir/Pibrentasvir 100/40 mg  Glecaprevir/Pibrentasvir 50/20 mg/pellets | Glecaprevir compound | Filed |  |
|  | Pibrentasvir compound | Filed |  |
|  | Glecaprevir/Pibrentasvir solid compositions I | Filed |  |
|  | Glecaprevir/Pibrentasvir solid compositions II | Filed |  |
| Gilead Sofosbuvir+Daclatasvir 400+60 mg  Sofosbuvir/Daclatasvir 400/60 mg | Sofosbuvir active metabolite | Filed |  |
|  | Daclatasvir compound | Withdrawn |  |
|  | Sofosbuvir compound (prodrug) | Filed (opposed) |  |
|  | Daclatasvir crystalline forms | Withdrawn |  |
|  | Sofosbuvir processes, intermediates & product-by-process | Filed |  |
|  | Sofosbuvir crystalline forms & preparation processes | Filed (opposed) |  |
|  | Sofosbuvir processes & intermediates | Filed |  |
|  | Compositions comprising crystalline Sofosbuvir | Filed (opposed) |  |
| Gilead Sofosbuvir/ledipasvir 400/90 mg | Sofosbuvir active metabolite | Filed |  |
|  | Sofosbuvir compound (prodrug) | Filed (opposed) |  |
|  | Ledipasvir compounds | Filed |  |
|  | Sofosbuvir processes, intermediates & product-by-process | Filed |  |
|  | Sofosbuvir crystalline forms & preparation processes | Filed (opposed) |  |
|  | Sofosbuvir processes & intermediates | Filed |  |
|  | Sofosbuvir in combination with Ledipasvir or Radalbuvir compositions | Filed (opposed) |  |
|  | Compositions comprising crystalline Sofosbuvir | Filed (opposed) |  |
| Gilead Sofosbuvir/Velpatasvir 150/37.5 mg/pellets  Sofosbuvir/Velpatasvir 200/50 mg/pellets  Sofosbuvir/Velpatasvir 400/100 mg | Sofosbuvir active metabolite | Filed |  |
|  | Sofosbuvir compound (prodrug) | Filed (opposed) |  |
|  | Sofosbuvir processes, intermediates & product-by-process | Filed |  |
|  | Sofosbuvir crystalline forms & preparation processes | Filed (opposed) |  |
|  | Sofosbuvir processes & intermediates | Filed |  |
|  | Velpatasvir compounds | Filed |  |
|  | Compositions comprising crystalline Sofosbuvir | Filed (opposed) |  |
| Gilead Sofosbuvir/Velpatasvir/Voxilaprevir 400/100/100 mg | Sofosbuvir active metabolite | Filed |  |
|  | Sofosbuvir compound (prodrug) | Filed (opposed) |  |
|  | Sofosbuvir processes, intermediates & product-by-process | Filed |  |
|  | Sofosbuvir crystalline forms & preparation processes | Filed (opposed) |  |
|  | Sofosbuvir processes & intermediates | Filed |  |
|  | Velpatasvir compounds | Filed |  |
|  | Compositions comprising crystalline Sofosbuvir | Filed (opposed) |  |
|  | Voxilaprevir & combinations | Filed |  |

Adapted from Medspal <https://www.medspal.org/?countries%5B%5D=Thailand&disease_area%5B%5D=Hepatitis+C+(HCV)&page=1>

**Supplementary File 2: MPP licences**

| DAA | Patent holder | Licenced generic partners | Number of countries that can receive these medicines and percent coverage of global population |
| --- | --- | --- | --- |
| Daclatasvir (DAC) | Bristol-Myers Squibb (BMS) | Sublicences can be issued to qualified entities worldwide – currently Beximco Pharma, Cipla, Hetero, Laurus, Mylan, Natco and Zydus Cadila | 112 countries that are home to 65.4% of people living with HCV in low- and middle-income countries. |
| Glecaprevir/pibrentasvir (G/P) | AbbVie | Nonexclusive sublicences can be issued to any qualified entity in the listed countries and in India – currently Arene Lifesciences ltd, Mylan, Remington, USV | 96 countries accounting for 47.5% of the HCV burden worldwide. |
| Ravidasvir (RAV) | Pharco | Not specified. “Sublicensees will be selected by MPP between entities with willingness and capacity to manufacture the Licensed Compound and/or Licensed Products in a manner consistent with MPP’s Quality Policy. Sublicensees could be based anywhere in the world.” | 85.3% of people with hepatitis C in 139 low- and middle-income countries. |

Adapted from Medicines Patent Pool <https://medicinespatentpool.org/progress-achievements/licences#Viral-Hepatitis>
